# Supplementary material for: A Multi-Medium Analysis of Human Health Risk of Toxic Elements in Rice-Crayfish System: A Case Study from Middle Reach of Yangtze River, China
Source: Foods. 2022 Apr 16;11(8):1160. doi: 10.3390/foods11081160 (PMC9024938; doi:10.3390/foods11081160)
Supplement: Supplementary file 1 [file foods-11-01160-s001.zip › foods-1658552-supplementary.pdf]

Supplementary Material

A multi-medium analysis of human health risk of heavy metals in rice-crayfish system, a case study from middle reach of Yangtze River, China

Hui Zhou [a1], Tao Ge [b1], Hui Li [a] *, Ting Fang [c], Huaiyan Li [a], Yanhong Shi [a], Rong Zhang [a], Xinju Dong [d]

[a] Anhui Province Key Laboratory of Farmland Ecological Conservation and Pollution Prevention, College of Resources and Environment, Anhui Agricultural University, Hefei 230026, Anhui, China

[b] Anhui institute of Geological Experiment, Hefei 230001, Anhui, China.

[c] Key Laboratory of Freshwater Aquaculture and Enhancement of Anhui Province, Fisheries Research Institute, Anhui Academy of Agricultural Sciences, Hefei 230031, Anhui, China

[d] Department of Chemistry, University of Louisville, Louisville, Kentucky 40292, USA.

* Corresponding author.

E-mail address: zhpaper@126.com (Hui Li)

[1]These authors contribute equally to this work.

List of Figures and Tables

Table S1. Instrumental operating conditions for the analysis of elements.

Table S2. The variables input for Monte Carlo simulations.

Table S3. The total variance explained and component matrices for the heavy metals in soils from RCS.

Table S1. Instrumental operating conditions for the analysis of elements.

**Microwave digestion**	**Successive step**	**Heating program**
	Step (1)	6 min, 120 °C, maintained 3 min
	Step (2)	5 min, 160 °C, maintained 3 min
	Step (3)	5 min, 190 °C, maintained 25 min
**ICP-OES**	**Parameters**	**Value**
	Plasma power	1300W
	Pump speed	30 rpm
	Coolant flow	14 L/min
	Auxiliary flow	1.0 L/min
	Nebulizer flow	0.7 L/min
	Optical temperature	14.0-16.0 °C
	Nebulizer pressure	2.0-4.0 Bar
	Main Argon Pressure	6.0-8.0 Bar
	Sample uptake rate	0.8 L/min
	Replicates	3

Table S2. The variables input for Monte Carlo simulations.

Variable			Distribution	Unit
Body weight (BW)		Adults	Lognormal (Mean=70, SD=5.8)	BWkg
		Children	Lognormal (Mean=25, SD=5.7)	BWkg
Ingestion rate (IR)	Rice	Adults	Lognormal (Mean=0.34, SD= 1.2)	kg/day
		Children	Lognormal (Mean=0.24, SD= 1.8)	kg/day
	Crayfish	Adults	Lognormal (Mean=0.055, SD=1.4)	kg/day
		Children	Lognormal (Mean=0.02, SD=2.1)	kg/day

Table S3. The total variance explained and component matrices for the heavy metals in soils from RCS.

Component	Initial Eigenvalues			Extraction Sums of Squared Loadings		
	Total	% of Variance	Cumulative %	Total	% of Variance	Cumulative %
1	5.711	71.387	71.387	5.711	71.387	71.387
2	0.926	11.570	82.958			
3	0.743	9.285	92.243			
4	0.277	3.460	95.703			
5	0.177	2.208	97.911			
6	0.092	1.153	99.064			
7	0.053	0.657	99.721			
8	0.022	0.279	100.000			

Metals	Component Matrix [a]
	**PC1**
Zn	0.976
Ni	0.973
Cr	0.943
Cu	0.941
Pb	0.905
As	0.857
Cd	0.512
Hg	0.470

Note: Extraction method: principal component analysis. [a] one component extracted.
